# Reply to the Comment of G. Feulner

**DOI:** 10.1002/andp.201100171

**Published:** 2011-09-20

**Authors:** Werner Weber

**Affiliations:** Institut für PhysikTU Dortmund, 44221 Dortmund, Germany

**Keywords:** Climate, terrestric solar irradiance, solar activity, data reduction

## Abstract

In my reply I present a re-analysis of the data of the Smithsonian Astrophysical Observatory (SAO). For this, a new data reduction method is introduced, allowing a drastic lowering of data scatter, so that the time series of the reduced data clearly shows the ≈ 1% variation of the terrestric solar irradiance in parallel with solar activity. The implications are discussed.

## 1 Introduction

**Fig. 1:**
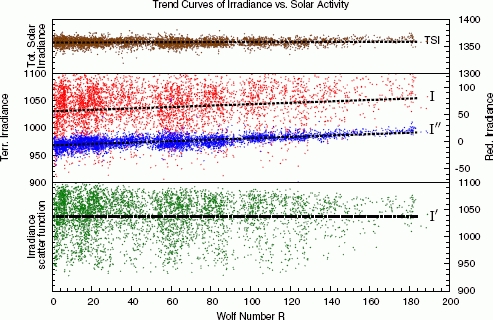
(online colour at: http://www.ann-phys.org) SAO Mt. Montezuma *M_O_* = 2.0 data of ‘precipitable water’ *W* (aqua) and of diffuse scattering (aureole *A*, green) vs. terrestric solar irradiance *I*. Second order trend lines are indicated. Inset shows plot of aureole *A* vs. precipitable water *W*, with a linear trend line. Note that the aureole data start around 4 W/m^2^, as emphasized in the inset by the dotted line. The area below 4 W/m^2^ indicates the contributions from N_2_ and O_2_ Rayleigh scattering.

Georg Feulner starts his comment [1] pointing out that modulations of the terrestric solar irradiance of order 1%, as presented in my paper [2], are incompatible with the observed global temperature variations. His claim is based on the results of climate model calculations (see his Fig. 1 and references in [1]). He then argues that the SAO data (and probably also the Mauna Loa data analysed in [2]) are fouled up by volcanic eruptions and, towards the end of the SAO data series, possibly by anthropogenically generated aerosols. In a previous paper [3], where he has presented a detailed critique of my paper, he has pointed out some more problems of the SAO data. He has observed, e.g.; that the distribution of measuring days is uneven. Periods of many consecutive measuring days alternate with periods of scarce data taking. Would this not lead to systematic errors? Also, in his analysis, the standard construction of ‘anomalies’ from the data has led, in some cases, to significant changes in the dependence of trends on solar activity. The anomaly construction removes, by subtracting monthly means, the annual cycle from the data and thus should not influence long term trends. He also points out volcanic activity during the minimum between solar cycles 16 and 17 (around 1931) which in his opinion deepened the irradiance minimum and thus feigned a strong dependence on solar activity.

In the following, details of the SAO measurements are discussed. These details suggest a new way for an improved data analysis. Application of the new method shows that my original results stand all critique.

## 2 Improved data analysis

The objective of the SAO project, set by Langley in 1905, was to measure by terrestric means the solar constant (total solar irradiance, TSI) at the top of the atmosphere [4]. The first idea was to measure by pyrheliometry the terrestric solar irradiance *I* at various zenith angles α or “optical masses” *M*_O_ = 1/cos α. As *M*_O_ ≈ *L*, the length of the solar path through the atmosphere, an extrapolation to *L* = 0 would yield the TSI value (Langley method). However, it was realized that diurnal variations of the data severely influence this extrapolation. Therefore, additional data were taken to include information of the atmospheric transmission. These data comprise the aureole *A*, the diffuse scattering in an angle of 15° around the solar beam, measured by pyranometry, and the ‘precipitable water’ *W*, the water content of the atmosphere, measured by bolometry at various ‘water lines’ and at ‘normal’ bands of the solar spectrum. The latter procedure was quite demanding, as it had to be executed in the short time span around a specific zenith angle or optical mass. The studies, how to optimize the correction of the *I* values by the *A* and *W* values, lasted between 1905 and 1923. They are partly described in Fowle's paper series 1912–1915 [5], where Fowle also presented his famous estimate of Avogadro's number from Rayleigh scattering. As a result, SAO developed a second method (‘short method’) to extrapolate to the TSI value from one terrestric solar irradiance data point *I_i_* at one specific value of *M*_O_. The precise way how this was done is not given in the literature. In the SAO data taken between 1923 and 1954 [6], there is a column ‘Function’, where the extrapolation value (or an intermediate step) is given.

The remarkable result is that SAO has determined TSI to 1357 W/m^2^ (at low solar activity), while the most modern satellite data [7] put it to 1361 W/m^2^, replacing the ACRIM value of 1368 W/m^2^, see, e.g.; [8]. Furthermore, even the small change of TSI with solar activity by ≈ 0.1% is given correctly, within the error bars [6].

This is the more surprising, as the data for *I*, *A*, and *W* show a much stronger dependence on solar activity, of order ≈ 1% and more, positive with solar activity for *I*, and negative for *A* and *W* (the latter only for one of the two main measuring sites, i.e., Mt. Montezuma in the Atacama desert near Calama, Chile; for the site Table Mountain near Los Angeles, no significant dependence of *W* on solar activity was found). Furthermore, the scatter of the extrapolated TSI values is much smaller than the scatter in the *I*, *A*, and *W* data (see also below).

In Fig. 1 the strong and nonlinear correlations between the terrestric solar irradiance *I* and the aureole *A* and water content *W* are shown. On the other hand, *A* and *W* show, to a good approximation, a linear correlation. It is surprising that for small values of *W* or *A*, any increase in *W* or *A* strongly reduces *I*. It means, e.g., that for small atmospheric water concentrations the *I* reduction is much more significant than for large water concentrations. How can that happen? The *I* reduction is either due to absorption at the water lines of the solar spectrum or due to scattering related to atmospheric water. Absorption by molecular water should be linear in water vapor concentration. However, the linear part seems to occur only at larger water concentrations. Similarly, the diffuse scattering related to atmospheric water should be caused by Rayleigh scattering of water molecules. This is consistent with the observation that the aureole values *A* increase linearly with *W*. However, the absolute value of the Rayleigh scattering of a water molecule is comparable to that of a O_2_ or N_2_ molecule. As we have atmospheric water concentrations of order 1%, the Rayleigh scattering of water vapor should be very small.

**Fig. 2:**
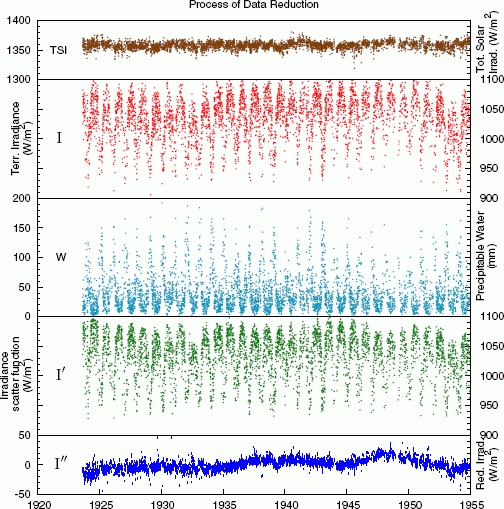
(online colour at: http://www.ann-phys.org) Top graph: Top of atmosphere total solar irradiance (TSI) series (red) as obtained by SAO for the data Mt. Montezuma *M*_O_ = 2.0. Second graph: Terrestric solar irradiance data *I* (red) for *M*_O_ = 2.0. Third graph: ‘precipitable water’ *W* (aqua), anti-correlated to *I*. 4th graph: *R*-independent scatter function *I*′ (green) which contains most of the annual variability of *I*. Bottom graph: time series of reduced terrestric irradiance *I*″(*R*), which exhibits much less scattering than original data irradiance *I* (2nd graph). Note that all irradiance plots have the same scale.

The unusual absorption and scattering of atmospheric water has been pointed out already by Fowle hundred years ago [5], but has not been resolved ever since. Fowle then had suggested that water carrying aerosols could produce the extra absorption (also at water lines) and the extra scattering. These aerosols may be produced by cosmic rays (which provide the condensation nuclei), but also by evapotranspiration of plants. These aerosoles carry only a fraction of the atmospheric water, but absorption and scattering strengths may be much bigger for aerosoles than for water vapor of the same mass.

The strong correlation between *I*, *W* and *A*, as evident in Fig. 1, also shows up in Fig. 2. Here, the variation in time of irradiance *I* and atmospheric water *W* is compared. Clearly, any increase of *W* is paralleled by a decrease of *I* – this strongly suggests that an increase of *W* is causing the decrease of *I* and may also cause the increase of *A*. So we can view the series *I_i_* as a function of the series *W_i_* and *A_i_* (where the *A_i_* can also be seen as a function of *W_i_*). Furthermore, all the quantities *I_i_*,*W_i_*, and *A_i_* depend on solar activity, represented by the sunspot or Wolf numbers *R_i_*.

Thus we have *I* = *I(W, A, R)*, or more precisely *I* = *I(W(R), A(W(R), R), R)*. Obviously, the scatter in the irradiance data *I* is mainly produced by the variations in the data *W* and *A*. Therefore, we try to generate a irradiance scatter function *I*′ = *I*′ (*W, A*) which contains most part of the data scatter. Then the difference of the two quantities *I* and *I*′ leads to a new function, the reduced irradiance *I*″; = *I(W, A, R)* − *I*′ (*W, A*) which should exhibit much less data scattering.

The simplest way is to generate a scatter function *I*′, which depends linearly on *W* and *A*. Yet Fig. 1 indicates that quadratic dependences are needed, in addition. When we have found *I*′ (*W, A*), there remains the problem that *I*′ depends on the solar activity, i.e., *I*′ (*W, A*) = *I*′ (*R*), via the *R* dependence of *W* and *A*. To remove the *R* dependence in *I*′ we use a Legendre-type transformation to the *R*-independent irradiance scatter function 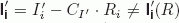. Here *C*_*I*′_ is the slope of the trendline of *I*′ and *R*, and can be seen as the first derivative of *I*′ with respect to *R*. The same transformation can be applied to *W* → **W_i_** = *W_i_* − *C_W_* ċ *R_i_* and to *A* → **A_i_** = *A_i_* − *C_A_* ċ *R_i_*.

**Fig. 3:**
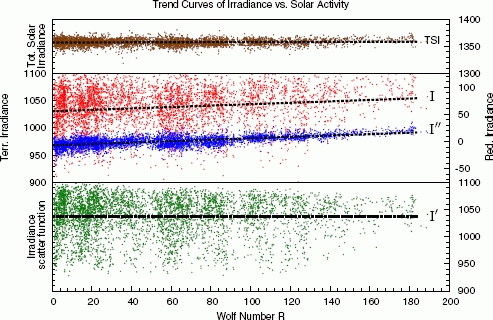
(online colour at: http://www.ann-phys.org) Trend analysis of TSI, of terrestric irradiances *I* and *I*′, and of the irradiance scatter function 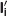, all versus 100 days averaged sunspot numbers *R*, again for *M*_O_ = 2.0, Mt. Montezuma. All plots have the same scale. Note the precisely parallel slopes of *I* (red) and *I*″ (blue) in the middle graph, and, in the bottom graph, the slope of *I*′ which is zero by construction. For comparison, in the top graph, the corresponding SAO-TSI data are shown. Note the very small slope there.

The irradiance scatter function *I*′ = *I*′ (*W, A*) is obtained by constructing a power series


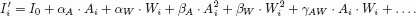


Here, the coefficients *I*_0_, α_**A**_, α_**W**_, β_**A**_, β_**W**_, γ_**AW**_ are found by requiring 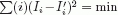. This leads to a set of linear equations involving higher order variants and covariants of the data sets involved. After the transformation 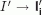, the *R*-independent scatter function 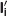 yields the reduced terrestric irradiance 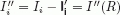. 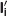 and *I*″ are also shown in Fig. 2. As can be seen, *I*″ exhibits a strongly reduced data scattering. Figure 3 demonstrates that the *R* dependence of *I*″ is the same as that of *I*: the two data sets show identical slopes of their trend lines, while 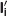 exhibits zero slope.

**Fig. 4:**
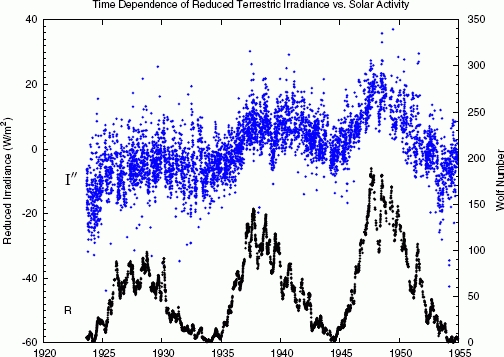
(online colour at: http://www.ann-phys.org) Time series of reduced irradiance *I*″ (blue) for *M*_O_ = 2.0 (Mt. Montezuma) in comparison with solar activity *R* (black). The *I*″ curve is centered at zero.

Figure 4 indicates that the time series of the reduced terrestric irradiance *I*″ indeed shows variations in parallel to solar activity which are of order 10–20 W/m^2^ or ≈ 1% of the terrestric *I* values.

Now a word to volcanic activity. Figures 2 and 3 also show the respective TSI series of the SAO data, obtained from extrapolations based on the three series *I*, *W*, and *A*. The TSI series exhibits a similarly reduced data scattering as does our reduction method. Now let us assume atmospheric perturbances by dust aerosols, produced, e.g., by sand storms or volcanic eruptions. As a consequence, these perturbances would alter *I* and *A* by extra absorption and scattering of solar light. Yet *W* would not be changed. As a consequence, the extrapolation procedure would no longer yield TSI data in the very limited scattering range as shown in Fig. 2. It appears to be quite obvious that in those instances the SAO teams simply stopped taking data. This would explain the unusual distribution of measuring days. It would also explain that there is no signature of volcanic disturbances in the TSI data (In the Mauna Loa solar irradiance data, the volcanic signatures due to El Chichon and Pinatubo eruptions are dramatic, see, e.g. [9]).

Finally, we have demonstrated that any data reduction procedure must not alter the trend which is searched for. As the annual cycle of *I* is mainly produced by *W* and *A(W)*, it is not surprising that the ‘anomaly’ construction may change the trend on *R*, as actually seen in all data, yet with varying magnitude.

## 3 Closing remarks

In summary, I have tried to refute all points, which Georg Feulner has raised in his paper [3] and has summarized in his comment here [1]. Yet his critique has induced the methodological improvements presented here. In particular, I have tried to show that the terrestric solar irradiance data indeed do show variations of order 1%. This is also true for the Mauna Loa data, where another re-analysis has been carried out by us [10]. I am convinced that it will also show up when the solar irradiance data of the PANGAEA collaboration [11] covering solar cycle 23 are finally analysed.

It was pointed out by Georg Feulner that 1% or larger variations of terrestric solar irradiance are incompatible with the climate model results. This may indeed be the case. Here, it is not the place to start a debate on the quality of the results of climate model calculations. Yet, I may note that nature may provide an answer to this question in the next few decades. It has been announced recently by the solar science community that another solar activity minimum of the type of the 17th century Maunder minimum is imminent [12]. Thus for the next solar cycles to come, an inactive, ‘cool’ sun is to be expected. If the climate modellers were right, this would just lead to a small dent in the predicted catastrophic global warming. If not, we may see many cold winters for a long time to come.
